# The lived experiences of nurse preceptors in training new nurses in Qatar: qualitative study

**DOI:** 10.1186/s12912-023-01619-9

**Published:** 2023-12-04

**Authors:** Bejoy Varghese, Rida Moh’d Odeh A.M. AL-Balawi, Chithra Maria Joseph, Adnan Anwar Ahmad Al-Akkam, Albara Mohammad Ali Alomari, Esmat Swallmeh

**Affiliations:** 1 Neuroscience & Medical Department, In-patient services, Hamad General Hospital, Doha, Qatar; 2 Neuroscience Department, In-patient services, Hamad General Hospital, Doha, Qatar; 3 Neurosurgery, In-patient services, Hamad General Hospital, Doha, Qatar; 4 Department of Nursing and Midwifery, College of Health Sciences, Doha, Qatar; 5 Neuroscience, Medical & Outpatient Department, Hamad General Hospital, Doha, Qatar

**Keywords:** Nurse preceptors, New nurses, Experience

## Abstract

**Background:**

The role of preceptors is vital in the successful integration of new graduate nurses in hospital settings. This study aimed to explore the experiences of nurse preceptors in training newly joined nurses in Qatar.

**Methods:**

Qualitative study was conducted between May 2022 and May 2023. Online semi-structured interviews were conducted through MS Teams with 13 nurse preceptors who had completed preceptorship training and trained at least one newly joined nurse. Participants were recruited until data saturation was obtained and data were analyzed using qualitative thematic analysis.

**Results:**

The results of the study revealed several main themes: teaching strategies and progressive skill development in preceptorship, challenges faced by the preceptor and preceptor better supported in training new nurses. The preceptors utilized different techniques to support new nurses including demonstration, discussion, use of technology, application of real-life clinical scenarios, simulations, and a gradual decrease in supervision to promote independence. However, a significant challenge was also reported including preceptors experienced high levels of exhaustion from the dual responsibilities of training new staff while also performing their regular care duties.

**Conclusions:**

The study underscores the fundamental role preceptors play in the training and development of newly joined nurses. While the challenges are considerable, particularly related to managing workload, the sense of achievement following the successful completion of training a new nurse suggests a strong commitment to this role. Despite the challenges, preceptors demonstrated innovative strategies to ensure the successful development of their preceptees, highlighting the importance of preceptorship in nursing education and practice.

**Supplementary Information:**

The online version contains supplementary material available at 10.1186/s12912-023-01619-9.

## Background

Preceptors are multifaceted professionals, serving as educators, evaluators, protectors, and role models. They play an instrumental role in cultivating professional values, instilling confidence, honing goal-setting skills, and guiding clinical decision-making processes among their trainees [1, 2]. Nurse preceptors serve as crucial pillars in the hospital environment, particularly in terms of assimilating newly recruited nurses and fostering their job satisfaction [3]. They also have a crucial part in integration and retention of newly recruited nurses [4]. With their vast experience and specialized knowledge in their respective fields, preceptors are effectively “educators” who offer on-the-job training to novice nurses and nursing students [5]. Leveraging their extensive experience and domain-specific knowledge, preceptors act as hands-on educators, facilitating the induction and training of novice nurses and nursing students [1]. Their primary role is to guide these new nurses in adjusting to the clinical environment and provide them with the specialized knowledge required for their job roles. Their pivotal role lies in assisting these new entrants in acclimatizing to the clinical environment and transferring specialized knowledge required for their professional roles [6].

The existing body of literature has documented the significant impact of preceptors on newly graduated nurses who are still in their early stages of professional development. [7]. Empirical evidence shows that regular interaction with preceptors significantly reduces stress among novice nurses [8, 9]. A study further highlighted this point by demonstrating that structured preceptorship programs aid in reducing novice nurses’ anxiety levels [10]. Moreover, a recent study conducted also underscores the effectiveness of preceptor-based training programs in enhancing the satisfaction and retention rates of newly recruited nurses [10]. Additionally, previous studies illustrated a positive correlation between the quality of care provided by preceptors and the job satisfaction and competence of new nurses [6, 11]. Such findings emphasize the importance of fostering healthy relationships between preceptors and novice nurses, ultimately leading to a more favorable perception of nursing work [1].

However, there is a significant problem we aim to address: there is limited research that explores the experience of preceptors in the workplace, creating a substantial gap in our understanding of their role and its impact on nursing education and patient care. This knowledge deficit hampers our ability to identify areas for improvement and provide better support for the crucial role that preceptors play in clinical education [12].

This study aims to bridge this gap by delving into the experiences of nurse preceptors in training newly joined nurses within the medical and Neuroscience units of a tertiary level hospital in Qatar. By doing so, it seeks to address the pressing issue of how to best prepare and support preceptors in their vital role, ultimately contributing to the objectives set forth in Qatar’s National Health Strategy 2018–2022. This strategy places a strong emphasis on the development of a skilled national workforce and the nurturing of a world-class healthcare system [13].

For the purpose of this study, “New nurses” encompasses a diverse range of individuals freshly entering the nursing job in the specific setting of Qatar’s healthcare system. This includes newly recruited nurses from overseas who, although they may have previous experience in their home countries, are novel to the dynamics of Qatar’s health system. It also comprises recent graduates from Qatari universities who have just commenced their practical careers after their educational journey [13]. Furthermore, it encompasses final practicum students from universities within Qatar who are transitioning from academia to full-time practical roles.

## Methods

### Design

This study utilizes a qualitative research method with a methodological orientation of phenomenology. Qualitative design allows for a comprehensive understanding of the lived experiences and perceptions of the participants [14]. The phenomenological approach underpins the study, enabling an in-depth exploration of the nurse preceptors’ experiences in training newly joined nurses. To ensure methodological rigor and transparency, this study follows the consolidated criteria for reporting qualitative research (COREQ) 32-item checklist [15].

### Setting

The study was conducted in medical and neuroscience units of a tertiary level hospital in Qatar.

#### Sampling method and recruitment

The study focused on the target population, which included a total of 109 nurse preceptors - experienced healthcare professionals who had successfully completed preceptorship training and were actively involved in training newly joined nurses. These nurse preceptors were identified and approached through email to participate in the study.

To ensure that the selected participants had the relevant experience and expertise, specific inclusion criteria were applied. Participants were required to meet two key criteria: (1) completion of preceptorship training, and (2) an experience of training at least one newly joined nurse. Nurse preceptors who did not meet both of these criteria were excluded from the study. This careful selection process ensured that the participants possessed first-hand experience and knowledge in the role of training newly recruited nurses within the context of inpatient neuroscience and medical units.

In line with the study’s objectives, a purposive sampling method was employed to select nurse preceptors with the necessary expertise and experience in the specific context of inpatient neuroscience and medical units [16]. This approach allowed for the deliberate and systematic selection of participants who could provide valuable insights into the experiences of nurse preceptors in this particular healthcare setting.

### Sample size

Out of the 109 nurse preceptors, a sample of 20 inpatient nurse preceptors were initially selected for the online interviews. However, during the course of the study, data saturation was reached after conducting interviews with 13 participants. Data saturation in qualitative research signifies the point at which new information or themes cease to emerge from the data [17]. Therefore, the sample size of 13 participants was deemed adequate for achieving the study’s research objectives.

### Data collection

Semi-structured, one-on-one online interviews were conducted through an online platform, MS Teams. The interviews were led by the study principal investigator, a male nurse/midwife educator with extensive nursing experience and a bachelor’s degree. The interview guide was specifically developed for this research to explore nurse preceptors’ experiences, challenges in the precepting process, and strategies for better support and fulfilment in the nurse preceptor role.

The online interviews were thoughtfully scheduled during nurses’ days off, allowing participants to engage in a calm and uninterrupted environment conducive to meaningful discussions, offering valuable insights, suggestions, and feedback. Before the interviews, participants voluntarily signed a research consent form, and the entire audio was meticulously recorded using MS Teams “Start Recording” feature. Verbatim transcripts were generated through the “Transcription” option in MS Teams.

Efforts were made to establish rapport and trust before the interviews. Participants were informed about the researcher’s professional background and motivations for conducting the research, ensuring transparency. The research team strictly adhered to confidentiality and secure data storage protocols. The interviewer maintained an unbiased, open stance toward diverse perspectives throughout the interviews.

Notably, there were no refusals to participate or dropouts, and only the participants and researchers were present during the interviews. The interviews continued until data saturation was reached, with audio-recorded interviews varying in duration from 29 to 43 min.

The interview guide underwent a pilot test before the main study to ensure the effectiveness and appropriateness of the interview questions and data collection process. The pilot test involved conducting trial interviews with a small sample of participants to identify any issues, refine the interview protocol, and make necessary adjustments to improve overall data collection quality. This pilot test was essential in adapting the interview guide to the unique context of qualitative research, where questions are often shaped by participant responses.

Field notes were taken during and after the interviews by the principal investigator. These notes captured important observations, reflections, and contextual details about interactions with the participants. Participants did not review or provide comments or corrections to the transcripts.

### Data analysis

The thematic analysis framework described by Braun and Clarke (2006) was employed to explore the lived experiences and perceptions of preceptors in training new staff and the challenges they encountered, as well as the value they placed on their role in training new nurses [18].

In this study, the verbatim data collected from in-depth interviews were transcribed using the “Transcription” option in MS Teams. The transcripts were then anonymized by assigning pseudonyms to the participants, and they were not returned to them for comments. No software was used for coding in this study. Thematic analysis involved six distinct steps. Initially, two authors (BV and CJ) engaged in the process of familiarizing themselves with the data by reading the transcripts multiple times. During this phase, quotes were classified and clustered into themes.

Throughout the analysis, two researchers (BV and CJ) independently reviewed and coded the interview transcripts, identifying initial themes and subthemes. To enhance reliability and agreement, a third researcher (AA) was involved in developing credibility around the themes and addressing any discrepancies between the initial coders. Following the reconciliation process, the final codebook was revised, and clusters of linked codes were organized into categories, emergent themes, and supported by verbatim quotes. Participant quotations were effectively utilized to illustrate the themes and findings, and each quotation was attributed to the corresponding participant number. The themes were iteratively refined and revised to ensure credibility and trustworthiness among the researchers.

### Trustworthiness of qualitative data

All elements of trustworthiness, including credibility, dependability, and transferability, were carefully considered in this study [19]. Credibility was attained through the sustained engagement of participants and the application of the researcher’s professional expertise. To enhance dependability, comprehensive records of the research process were maintained, facilitating the potential replication of similar research endeavors. In terms of transferability and confirmability, detailed journal notes were diligently kept, and a comprehensive audit trail of all research processes was meticulously maintained.

## Results

### Preceptor characteristics

A total of 20 inpatient nurse preceptors were initially selected for the study from a pool of 109 nurse preceptors, but data saturation was reached after interviewing 13 participants during the course of this research. The average age of the preceptors was found to be 38.6 years, whereby more than half of them, (54%), were observed to be within the age range of 30–40 years. All the preceptors were female. More than half of participants have a Bachelor of Science in Nursing (BSN) degree (54%) and 46% have a diploma.

The mean number of years of experience was 14.9 years. In terms of their experience as preceptors, the mean number of years of preceptorship experience was 7.4 years. The characteristics of the preceptors participating in the study are summarized in Table 1.

**Table 1:** Preceptor characteristics (*N*=13)

Variable	n	%
Age		
30-40	7	54
41-50	6	46
Gender		
Male	0	0
Female	13	100
Educational Level		
Diploma	6	46
BSN	7	54
Specialty of work		
Medical	9	69
Neuroscience	4	31
Nursing Experience in HMC (years)		
0-10	4	31
11-20	9	69
Preceptor experience(years)		
0-5	4	31
6-10	9	69

Three main themes emerged from the qualitative analysis: (1) Teaching Strategies and Progressive Skill Development in Preceptorship (2) Challenges faced by the preceptor, and (3) Preceptor better supported. Table 2, provide a summary of the themes. Preceptors are marked from P1 to P13.

**Table 2:** Preceptors’ new nurse’s training experiences

Themes	Subthemes
Teaching Strategies and Progressive Skill Development in Preceptorship	1. Preceptor teaching strategies
	2. Practical opportunities and hands-on experience
Challenges faced by the preceptor	1. Overload of Roles and Responsibilities
	2. Language and Cultural Barriers
	3. Lack of Support and Appreciation
Preceptor better supported	1. Recognition and Appreciation
	2. Personal and Professional Growth
	3. Trust in the Role of Preceptor

## Teaching strategies and progressive skill development in preceptorship

The theme of “Teaching Strategies and Progressive Skill Development in Preceptorship” emerged from the narratives of nurse preceptors who shared their experiences in training newly joined nurses in medical and neuroscience units of a tertiary hospital in Qatar. Within this theme, preceptors detailed their innovative teaching strategies, emphasizing personalized approaches that catered to the unique needs of their preceptees. Their wealth of experience and domain-specific knowledge allowed them to implement diverse learning methods, fostering a dynamic learning environment. This theme highlights the critical role preceptors play in shaping the skill development of novice nurses through hands-on guidance and mentorship.

### Preceptor teaching strategies

Preceptors highlighted a range of teaching strategies they employ in their role, focusing on hands-on instruction, demonstration and re-demonstration, and a step-by-step progression of learning objectives.

Many preceptors approach the teaching process with a gradual progression of skills. They start with basic tasks and as the preceptees gain confidence and competence, they move on to more complex responsibilities. This not only helps preceptees gradually acclimate to their new roles, but also gives them the opportunity to build a solid foundation before moving on to more complex tasks. This is encapsulated in the P1 quote:

“*First week, I won’t teach anything, only vital signs checking, NGT feeding and personal care. After that medication exam. First my preceptee will watch while I am demonstrating and will re-demonstrate. Once I will feel my preceptee confident, they can do themselves”.*

The teaching method of demonstration followed by re-demonstration is frequently utilized by the preceptors. They believe it allows for a hands-on approach where preceptees can observe a task being done correctly before they have to perform it themselves. This method reinforces learning, ensures competency, and builds confidence. As P2 explains “*I used observation, direct instruction, discussion, lecture, sharing clinical personal experience, active listening and embedded strategy as my way of teaching*”.

### Practical opportunities and hands-on experience

Developing skills through practical opportunities and hands-on experience was highlighted by the preceptors. They strive to provide their preceptees with real patient scenarios, case presentations, and simulations to bridge the gap between theoretical knowledge and clinical practice.

Preceptors place a high value on ensuring that preceptees get exposure to real clinical situations. P3 believes that nurses “*will learn if there is only real situation. I am directing my preceptee to my colleagues for any new procedure*”. Such experiences also help preceptees in developing problem-solving skills and thinking critically in high-pressure situations. Preceptors frequently use simulations and case presentations in their teaching strategies. These methods allow preceptees to apply theoretical knowledge in a simulated clinical scenario, facilitating their understanding of how different pieces of knowledge fit together in-patient care. P4 says: *“We will use case presentation and simulation which will help my preceptee to correlate their theoretical knowledge with practical”.*

Preceptors recognize the significance of fostering self-sufficiency and instilling confidence in their preceptees while simultaneously cultivating a supportive and nurturing learning environment that promotes questioning, evaluation, and learning from mistakes. To achieve this goal, they gradually stops supervision, allowing preceptees to progressively assume more autonomy. The preceptors gradually reduce their level of supervision to inspire self-sufficiency and self-assurance in their preceptees. As preceptees become more skilled and knowledgeable, they are given more responsibility, which helps them to become self-sufficient and self-reliant practitioners as P1 explains: “After two weeks I will step back little by little to see how much they can manage independently”.

Preceptors strive to create a supportive learning environment where they are “*always open for any questions and doubts nurses have and will discuss with them to make their concept clear*” according to P5. Preceptors believe that such an environment helps preceptees to learn effectively, build confidence, and develop their problem-solving skills.

Feedback is seen as a crucial element in the learning process by the preceptors. Preceptors attempts to continually give timely, non-judgmental feedback, highlighting both strengths and areas needing improvement. P6 believes that *“Timely and constructive feedback, delivered without judgment, and accompanied by clear explanations of both strengths and areas for improvement, can motivate preceptees to evaluate and discuss their performance. This approach enhances educational strategies that promote critical thinking and a commitment to excellence”.*

Preceptors use observation as a primary method to assess the learning progress of their preceptees. Watching preceptees perform tasks allows them to evaluate the understanding and application of theoretical knowledge in a practical setting. They also encourage preceptees to reflect on their daily activities and experiences, fostering a continuous learning environment. P7 explains this as: *“I ensure that my orientees are learning from me by direct questioning, discussion and reflection from the daily activities”.*

## Challenges faced by the preceptor

The theme of “Challenges Faced by the Preceptor” originates from the candid reflections of nurse preceptors as they navigated the complexities of their role. These challenges encompassed the dual responsibilities of training new staff while maintaining their regular patient care duties. The origin of this theme lies in the recognition of the significant burden and exhaustion experienced by preceptors due to the demanding nature of their role. This theme underscores the need to address these challenges to ensure the well-being and effectiveness of nurse preceptors.

### Overload of roles and responsibilities

While preceptors are committed to providing high-quality patient care, they are also tasked with the role of a preceptor, responsible for guiding and mentoring new nurses. The administrative duties that come along with these roles often pile up, creating a sense of being overwhelmed. The challenge is thus to efficiently manage time and tasks, ensuring that neither patient care nor mentoring duties are compromised, all while dealing with the bureaucratic aspects of their roles.

Preceptors face the challenging task of balancing their responsibilities for patient care and mentorship, which can put a strain on their abilities. Attempting to devote equal time and effort to both roles can result in feelings of frustration and burnout. This challenge is made even more difficult by the unpredictable nature of healthcare settings, where unforeseen circumstances and emergencies can add complexity. P8 says *“I remember a particularly intense situation when a patient’s condition suddenly deteriorated, and I had to guide the new nurse on how to respond quickly and effectively. It was like a test of our training and readiness. That experience taught me the importance of being prepared for the unexpected in healthcare”.*

In addition to caring for patients and mentoring, preceptors must also handle significant administrative tasks. This includes *“Paperwork is overload”.* The bureaucratic aspect of their role often demands time and effort that could otherwise be spent on patient care or mentoring. The difficulty is not only the amount of paperwork, but also the fact that these tasks frequently must be completed “*After regular duty hours*”, cutting into personal time as per P9.

### Language and cultural barriers

The theme encompasses the issues that arise when preceptors must interact with trainees from diverse cultural and linguistic backgrounds. Preceptors stated that communication forms the backbone of effective preceptorship. However, P10 recalls “Some staffs, they have a language problem and training commonly difficult because of new non-English speaking staffs”. They reported that this led to misunderstandings and slow down the learning process, causing frustration on both ends*.*

P9 says “*when trainees come from various countries and backgrounds, they have their own learned practice and experience that they are continuously using in the corporation, they have different perceptions and not open for changes in skills and practice*”. This diversity may create clashes at times with existing procedures and guidelines. The preceptor, therefore, has the additional task of reconciling these differences and molding the preceptees according to the current practices. P9 says “*most of the experienced nurses from different region*”.

### Lack of support and appreciation

The preceptors expressed a sense of disappointment regarding the inadequate support and recognition they receive in their role. It is common for preceptors to take on additional responsibilities, and when these efforts go unnoticed and unrewarded, it can lead to feelings of dissatisfaction and demotivation. The preceptors requested collegial support to establish a precepting-friendly environment and according to P10 “*When colleagues and superiors are empathetic and encouraging, it can significantly ease the burden of our job*”. However, the absence of such support can exacerbate the challenges associated with fulfilling the precepting role and contribute to stress. *“I feel like the absence of support makes everything so much harder. The demands of precepting can be overwhelming, and when you don’t have the support you need, it just adds to the stress and makes it really difficult to do our job effectively as per P11.“*

There is a feeling among preceptors that their efforts are not adequately acknowledged or rewarded. This can significantly impact their morale and willingness to take on extra responsibilities, potentially affecting the overall quality of preceptorship.

*P13 says “I feel pressurized by additional job without any incentives and appreciation”.*

## Preceptor better supported

The theme of “Preceptor Better Supported” emanates from the narratives of nurse preceptors who highlighted the importance of continued support and resources in their role. Their experiences underscored the critical need for ongoing professional development, mentorship programs, and recognition for their contributions. The origin of this theme lies in the collective voice of preceptors advocating for improved support systems to enhance their effectiveness in training newly joined nurses. This theme emphasizes the importance of investing in preceptorship programs to facilitate the successful integration and retention of novice nurses, ultimately benefiting patient outcomes and the overall healthcare environment.

### Recognition and appreciation

Many of the participants express a feeling of contentment when their work as mentors is acknowledged, whether verbally or formally. The mentors note that recognition, especially in the form of appreciation from those in higher positions, boosts their motivation and satisfaction. Official recognition would not only validate their hard work but could also be added to their professional portfolios, which could help advance their careers. P3 explains “*If I received a certificate of appreciation, that would be great - I could keep it in my portfolio*”. The mentors acknowledge how even a simple act of verbal recognition affects their morale as one preceptor explained, “*When preceptee is happily telling that they learned something new…. it will make my day*”.

### Personal and professional growth

The preceptors perceive the role as an opportunity to improve their professional skills and update their knowledge. They believe that teaching others helps them revise and consolidate their own understanding of nursing science. P9 says “*Being a preceptor is more than just a responsibility; it’s a chance to increase my nursing knowledge and improve my own skills. Being a preceptor contributes to the development of my competencies*”. They also highlight how they benefit from being continually updated on the policies and practices of their healthcare setting. P8 affirms, “*I am updating the knowledge from the HMC policies and procedures while training new staff*”.

P8 stated that being a preceptor increases “*confidence after each training, as I gain more knowledge*”. The role requires guiding and mentoring trainees, leading to an enhancement of these traits over time.

### Trust in the role of preceptor

Preceptors viewed the frequent assignment of new trainees as an expression of trust in their abilities. This strengthens their sense of being valued and trusted in their role as P12 explained “*Yes, I feel trusted in the role as a preceptor. I have completed the tasks assigned to me, so the HN is giving me preceptee again and again”.*

Experienced preceptors who have served for several years and mentored multiple trainees perceive their extensive tenure as evidence of the trust vested in them. They believe that their ability to successfully guide numerous trainees over time reflects the confidence their superiors have in their precepting abilities. As P13 expressed, *“Yes, that’s why I had 5–6 preceptees”.*

## Discussion

The aim of this study was to explore the experiences of nurse preceptors in training newly joined nurses in the medical and neuroscience departments. The findings from the interviews conducted with the preceptors shed some light on the role and strategies used by the preceptors to perform their jobs. However, the results revealed that preceptors have many challenges and in order to face these challenges, preceptors identify few strategies to continue supporting the new nurses.

Clinical preceptors in the present study employ diverse techniques to facilitate the learning and development of newly joined nurses who come from different backgrounds and possess distinct learning requirements. Nursing is a profession that attracts individuals from diverse backgrounds, each with their own unique learning preferences [20]. Given the diverse backgrounds of these nurses, preceptors in this study employ a variety of teaching strategies that may enable them to cater to the various learning styles and preferences of the nurses [21]. By employing multiple teaching strategies, preceptors can assist new nurses in refining their critical thinking skills, enhancing their ability to prioritize patient care, and improving their decision-making skills in challenging situations. By providing ongoing guidance, preceptors can identify areas for improvement and offer constructive criticism to help new nurses improve their skills [22].

The findings of this study are in line with prior research, underscoring the crucial role of nurse preceptors in the education and training of newly joined nurses. The preceptors expressed a sense of fulfillment in their mentoring role and highlighted the significance of sharing their knowledge to foster the professional growth of novice nurses. These consistent results reinforce the existing evidence supporting the value of preceptorship programs in healthcare settings, underscoring the importance of providing adequate support and recognition to facilitate the successful integration and retention of newly recruited nurses [6, 22].

The satisfaction reported by the preceptors in this study is likely a result of feeling valued and recognized for their significant contribution to the training of new nurses. They expressed a sense of accomplishment from witnessing the growth and progress of their preceptees, which positively influenced their job satisfaction. These findings are consistent with existing literature, which highlights the importance of appreciation and recognition in the workplace. When preceptors feel acknowledged and valued for their efforts, it fosters a positive work environment, enhances their sense of purpose, and ultimately contributes to the successful integration and retention of newly recruited nurses [23]. Recognition and appreciation are essential elements in motivating and retaining experienced preceptors, ensuring their continued commitment to nurturing and guiding novice nurses. A strong and meaningful relationship between preceptors and nurses can influence the perception of nursing work and overall job satisfaction [1, 24].

However, despite the feeling of appreciation and being valued, preceptors are facing many challenges. The preceptors in this study reported experiencing significant exhaustion due to the added responsibilities of training newly recruited nurses in addition to their routine patient care duties. This finding is in line with previous research which also emphasized the demanding nature of the preceptor role [25]. The study indicated that preceptors often face the challenge of balancing their own workload while simultaneously providing guidance and support to novice nurses.

Moreover, the preceptors mentioned that the lack of adequate support and resources from the healthcare organization contributed to their feelings of exhaustion. They emphasized the need for additional support, such as workload management assistance and dedicated training time, to effectively fulfill their preceptor roles. The absence of such support could potentially hinder the preceptors’ ability to provide optimal guidance and training to the newly recruited nurses [23]. Our findings resonate with the research which also highlighted the challenging nature of the preceptor role [26]. The demands of precepting can impact not only the preceptors’ job satisfaction and well-being but also the quality of training provided to the novice nurses [26]. Therefore, addressing the challenges faced by preceptors and providing them with the necessary support and resources is crucial to enhancing their effectiveness in training new nurses and ultimately improving patient care outcomes. To overcome these challenges, healthcare organizations should prioritize the implementation of comprehensive preceptorship programs, provide workload management support, foster a culture of appreciation and recognition, establish open communication channels, and invest in the professional development of preceptors [27]. By doing so, organizations can create a supportive environment that enhances preceptor effectiveness and contributes to a positive and sustainable healthcare workforce.

### Limitations

The study has several limitations. Firstly, it was conducted within a single department, which may limit the generalizability of the findings to other healthcare settings. Additionally, the lack of participation by male preceptors could introduce gender bias in the study’s insights. Furthermore, the subjective nature of qualitative data may be influenced by individual perspectives and biases, potentially affecting the objectivity of the study’s conclusions. It is crucial to acknowledge these limitations when interpreting the findings and considering their application in broader healthcare contexts.

## Conclusion

This study delves into the multifaceted experiences of nurse preceptors, highlighting their pivotal role in training newly joined nurses. The findings emphasize the necessity of ongoing support and resources for these professionals.

In summary, this investigation uncovers the invaluable contributions of nurse preceptors in facilitating the integration and retention of novice nurses, with potential benefits for patient outcomes and work environments. Challenges underscore the need for comprehensive support systems.

To conclude, recognizing and nurturing the role of nurse preceptors is vital. This research advocates for sustaining and enhancing existing preceptorship programs, fostering the development of support mechanisms, and empowering these professionals. Further research in this area is warranted to explore strategies for addressing the challenges faced by preceptors and enhancing the effectiveness of preceptorship programs.

### Supplementary Information

**Additional file 1: Supplementary File 1.** The Interview Guide.

## Data Availability

The availability of data and materials may be subject to certain access restrictions, such as ethical, legal or commercial sensitivities. The Corresponding author Bejoy Varghese at bejoy1987@gmail.com should be contacted if someone wants to request the data from this study.

## Abbreviations

COVID-19: Coronavirus disease 2019
HMC: Hamad Medical Corporation
IRB: Institutional Review Board
MRC: Medical Research Center
HGH: Hamad General Hospital
MoPH: Ministry of Public Health
