# Supplementary material for: The lived experiences of nurse preceptors in training new nurses in Qatar: qualitative study
Source: BMC Nurs. 2023 Dec 4;22:456. doi: 10.1186/s12912-023-01619-9 (PMC10694916; doi:10.1186/s12912-023-01619-9)
Supplement: Supplementary file 1 — Additional file 1: Supplementary File 1. The Interview Guide. [file 12912_2023_1619_MOESM1_ESM.docx]

**Supplementary File 1: The Interview Guide**

**A. Introduction**

Introduce moderator and co‐moderator and their role in this study research

**Moderator**: Preceptors are a key in retaining newly recruited nurses in a hospital setting. A preceptor is “an educator who gives on-the-job training to novice nurses and nursing students”. As experienced nurses with specialized knowledge in their area of work, preceptors can aid novice nurses in adjusting to the clinical environment and impart to them specialized knowledge needed in their work environment. The objective of this study is to explore the experiences of nurse preceptors working in Neuroscience and medical department of HGH in training newly recruited nurses. The participants of this qualitative study will be nurses who had completed preceptorship training, had experience training newly recruited nurses and are willing to share their precepting experience with the researcher. A semi structured in-depth one to one online interview through MS Teams will be done among **20 interested participants** out of total **109 nurse preceptors** working in Neuroscience and medical department of HGH.

**Moderator:** Thank you for agreeing to take part in this one-to-one online interview. We the team appreciates your willingness to participate.

The anticipated outcomes for this research project are,

- To understand the challenges of the nurse preceptors between their concurrent role as preceptors and nurses
- To identify the effectiveness of training new employee nurses in regard to extra workload, with patients given priority

Semi structured interviews will be conducted among 20 nurse preceptors who have trained any new employee nurses during their tenure in HGH. The nurses who wish to participate in interview were recruited through the link provided during the study.

**B. We have a few guidelines and rules to facilitate our interview:**

1. We want you to do explore your feelings on below questions. I may ask you to repeat if I haven’t heard from you in a while.

2. There are no right or wrong answers. All person’s experiences and opinions are important. We expect and want to hear a wide range of opinions and we do not anticipate consensus, just sharing.

3. We emphasize that what is said in this room should remain here. You should be comfortable to share anything if sensitive issues come up.

4. The interview will last for about one hour. Please silence your mobile phones.

We are only here to assist in this interview.

5. We will record this session as we want to capture everything you have to say. We don’t identify anyone by name in our findings. When you respond, be sure to not mention your name. You will remain anonymous. Your experiences and perception will be recorded and secured by the PI, Mr Bejoy Varghese. You can keep the video option on/off. We can provide summary details once the study is complete.

**C. Interview Questions**

1. Could you share your experience of being a nurse preceptor?

2. What challenges did you encounter in the precepting experience?

3. From your point of view, how could you be better supported to fulfil the nurse preceptor role?
